# Sulphite of Soda

**Published:** 1871-02

**Authors:** 


### THERAPEUTICS OF CHRONIC CONSTIPATION.

Dr. J. K. Splender, in a sensible paper in the *Medical Times and Gazette*, on the treatment of constipation, when it exists *per se* as a primary disorder of function, recommends the following judicious plan, which comprises four factors : (*a*) Minute and frequent doses of watery extract of aloes, not exceeding one gr. —very rarely, of extract of colocynth. (*b*) A dose of sulphate of iron; gr. jss. to gr. ij., always combined with the aperient. (*c*) Regulation of the diet. (*d*) Constitutional exercise. He does not believe that any other drug can supply the place of the sulphate of iron, though belladonna may be occasionally useful as an auxiliary. At first he gives a pill, composed as above, three times a day, immediately after the principal meals, and gradually reduces the number and frequency of the pills as the intestinal functions are restored. The action is slow, requiring two or perhaps three days, but nothing approaching to purgation ought ever to be permitted. As a substitute in certain cases, Mist. ferri co., with dec. aloes co., in corresponding doses, may be adopted. —*Medical and Surgical Reporter.*

---

### SULPHITE OF SODA.

Dr. G. Kelmer, of Illinois, writes to the *Western Journal of Medicine*, that sulphite of soda can be relied upon as a speedy and effectual remedy in almost all parasitic affections of the skin. "I have also used it in various other cutaneous diseases with uniform and unvarying success. And in that condition of the blood which is manifested by the production of numerous furuncles, commonly known as boils, the administration of sulphite of soda, with carminative tonics, has proved, under my observation, a perfect and rapid remedy. In carbuncles, I know that, after using the following, it may be relied upon. After a forced or spontaneous opening of the carbuncle, apply a solution, on lint, of, say :

℞—Sodæ sulphitis, . . . ℥ ss.

Acidi carbolici (crys.) . . ʒ ss.

Glycerine, . . . f. ℥ iij. M.

"It is remarkable how rapidly, under these applications, the ordinarily slow separation of the necrosed cellular tissues takes place—the destructive process ceases, and healthy granulations spring up.

"In uticaria it has proven very successful in subduing the worst forms of this disease in twenty-four hours. There are a great many other skin diseases in which I have found it equally efficient.

"Another application of this salt, which I consider valuable, is in the case of infants, by whom their food (the mother's breast-milk) is often ejected. A dose of two to five grains of sulphite, in combination with comp. tr. cardamom, sweetened, has proved successful in causing a retention and assimilation of the contents of the stomach when administered soon after imbibition, thus greatly promoting the health of the child. Also in cases of children where there is a fermented, swollen condition of the bowels, especially if constipated, the sulphite of soda will remove the difficulty in a short time."—*Drug Circular and Chemical Gazette.*

